# Cross-sectional evaluation of the periapical status as related to quality of root canal fillings and coronal restorations in a rural adult male population of Turkey

**DOI:** 10.1186/1472-6831-11-20

**Published:** 2011-06-20

**Authors:** Kaan Gündüz, Hakan Avsever, Kaan Orhan, Kadriye Demirkaya

**Affiliations:** 1 Department of Oral, Teeth and Jaw Radiology, Faculty of Dentistry, Ondokuz Mayis University, Samsun, Turkey; 2 Department of Oral, Teeth and Jaw Radiology, Gulhane Military Medical Academy, Ankara, Turkey; 3 Department of Oral, Teeth and Jaw Radiology, Faculty of Dentistry, Ankara University, Turkey; 4 Department of Endodontics, Gulhane Military Medical Academy, Ankara, Turkey

**Keywords:** Periapical status, rural, root canal treatment

## Abstract

**Background:**

To determine the prevalence of periapical lesions in root canal-treated teeth in a rural, male adult, Turkish population and to investigate the influence of the quality of root canal fillings on prevalence of periapical lesions.

**Methods:**

The sample for this cross-sectional study consisted of 552 adult male patients, 18-32 years of age, presenting consecutively as new patients seeking routine dental care at the Dental Sciences of Gulhane Military Medicine, Ankara. The radiographs of the 1014 root canal-treated teeth were evaluated. The teeth were grouped according to the radiographic quality of the root canal filling and the coronal restoration. The criteria used for the examination were slightly modified from those described by De Moor. Periapical status was assessed by the Periapical Index scores (PAI) proposed by Orstavik.

**Results:**

The overall success rate of root canal treatment was 32.1%. The success rates of adequately root canal treatment were significantly higher than inadequately root canal treatment, regardless of the quality or presence of the coronal restoration (P < .001). In addition, the success rate of inadequate root canal treatment was also significantly affected by the quality of coronal restorations.

**Conclusions:**

Our results revealed a high prevalence of periapical lesions in root canal treatment, which is comparable to that reported in other methodologically compatible studies from diverse geographical locations. In addition, the results from the present study confirm the findings of other studies that found the quality of the root canal treatment to be a key factor for prognosis with or without adequate coronal restoration.

## Background

There are plenty of longitudinal studies investigating the outcome of root canal treatment performed in educational hospitals (e.g., dental faculties), which have demonstrated that the reality for the overall population might be somewhat different, with only 35%-96% of the root canal treated teeth exhibiting no disease[1-5]. However, for the most part, epidemiological studies evaluating the outcomes of root canal treatments performed by general practitioners show much lower success rates. Frequencies of periapical lesions in root canal treatment have been reported in Belgium (40%), Denmark (52%) Lithuania (39%), Canada (44% and 51%), Germany (61%), Scotland (51%), Spain (64.5%), and The United States (39%)[6-9]. This discrepancy in success rates may reflect a difference in the quality of the root canal treatment performed despite improvements in instruments and materials and advances in the understanding of the disease process.

In cross-sectional studies, there is a consistent association between periapical radiolucency and inadequate root fillings [10,11]. Ray&Trope[12] reported that the quality of the coronal restoration had a greater impact on the periapical status of root canal treated teeth. In contrast to the findings of Ray&Trope[12], other studies have revealed that adequate root canal treatment had a greater influence than coronal restoration. Tronstad et al.[11] showed that the quality of a well-sealed coronal restoration only significantly influenced the outcome when combined with adequate endodontic treatment. The high rates of success reported in these studies were obtained with well-trained practitioners under strict operating condition that may not reflect the situation found within the average dental clinic [13].

Such studies demonstrate the potential outcome of root canal treatment rather than its realistic outcome in general practice [14].

In Turkey, 25% of the national population lives in a rural area. This study is the first to determine the prevalence of periapical lesions in root canal-treated teeth in a rural, adult, Turkish population and to investigate the influence of the quality of root canal fillings and coronal restorations on prevalence of periapical lesions.

## Methods

The sample for this cross-sectional study consisted of 552 adult male patients, 18-32 years of age, presenting consecutively as new patients seeking routine dental care at the Dental Sciences of Gulhane Military Medicine, Ankara. All these individuals were army recruits, who lived in different rural regions of Turkey. Any patient who lived in a city of Turkey was excluded from the study. An area with less than 10,000 inhabitants was classified as towns and villages (rural); and a population of more than 10,000 defined a location as a city. Another part of the inclusion criteria was that the patient had to be visiting the clinic for the first time.

To be enrolled in the study, the patient's chart had to contain a panoramic radiograph with supplemental periapical radiographs. Periapical radiographs were only used when panoramic radiographs showed poor image quality particularly in the upper and lower anterior region. All periapical radiographs were taken using the paralleling technique. All radiographs were taken and processed at the same radiology center in 2008. The Bluex Pantos 16 XP (Assago, Italy) panoramic roentgen unit and Trophy Trex X-ray (Croissy, Beaubourg, France) periapical radiography unit were used to take radiographs.

This study was based on retrospectively evaluation of radiographs. Thus, no ethical approval was obtained from the local ethical committee since only the achieve data were used for the study. However, before taking any radiograph or intra/extra-oral examination, patients gave their informed consent prior to radiography and examinations according to the principles of the Helsinki Declaration, including all amendments and revisions. Collected data was only accessible to the researchers. Moreover, all examiners in the study only examined the radiographs and were blinded to any other patient data in the radiographic examination procedure.

In total, the radiographs of 1014 root-filled teeth were evaluated. The teeth were grouped according to the radiographic quality of the root canal filling and the coronal restoration. The criteria used for the examination were slightly modified from those described by De Moor et al. [9] as follows:

### Quality of root filling

Adequate: All canals were obturated. There were no voids present. The end of the root canal fillings was 0-2 mm short of the radiographic apex.

Inadequate: The end of the root canal fillings were more than 2 mm short of the radiographic apex or grossly overfilled. Root canal fillings had voids, inadequate density, unfilled canals, and/or were poor condensation.

### Coronal restoration

Adequate: Intact restoration with good margin adaptation and no signs of recurrent caries

Inadequate Restoration with overhangs, open margins, recurrent caries; or no restoration at all.

### Periapical status

Apical status was assessed by the Periapical Index (PAI) proposed by Ørstavik et al.[15] who scored the apical area of the radiographic images as follows:

1. Normal periapical structures

2. Small changes in bone structure

3. Changes in the bone structure with little mineral loss

4. Periodontitis with a well-defined radiolucent area

5. Severe periodontitis with exacerbating features

A score of PAI 1 was defined as a healthy periapical region, indicating a normal width of the permanent ligament space and normal appearance of the surrounding bone. All other PAI scores were evaluated as apical periodontitis. All radiographs were evaluated independently by an endodontic consultant and a dental radiologist who both had over 10 years of clinical experience. Both examiners were calibrated. As a part of the calibration phase, study methodology was explained to the examiners. To minimize discrepant results they also familiarized themselves with the scores they should attribute to the radiographic images and the established evaluation method for the study. Two hundred teeth were assessed to calibrate the 2 examiners, and inter-examiner agreement was detected by Cohen's kappa (kappa = 0.86). The radiographs were observed using an x-ray viewer with 2 times magnification. To get optimal image quality, the room was darkened to control the surrounding light for the best radiographic contrast. When disagreement occurred, a third observer, a dental radiologist with 15 years of clinical experience, asked to make a decision.

SPSS software was used for statistical analysis (SPSS Inc., Chicago, IL). Differences between the groups were examined using the chi-square test. The significance level was established at 5%.

## Results

Of the 1014 root canal treatment investigated in this study from 552 male patients, maxillary central incisors were the most frequently treated teeth, followed by mandibular premolars, maxillary lateral incisors, and maxillary premolars (Table 1). Mandibular incisors were the teeth with the lowest frequency of endodontic treatment.

**Table 1:** The distribution of ROOT-FILLED treated teeth according to the type of tooth (n = 1014)

Tooth	Maxillary	Mandibular
Central incisor	225	20
Lateral incisor	162	24
Canine	123	31
Premolar	140	192
Molar	42	55
Total	692	332

On the basis of the PAI scoring system, 326 (32.1%) teeth were classified as healthy and 688 (67.9%) teeth as apical periodontitis. The root canal filling was rated as adequate in 425 teeth (41.9%). In this group, the success rate was 59.8%. The group with inadequate root canal treatment corresponded to 58.1% of the examined cases and had a success rate of 12.2% (Table 2). In general, the success rates of adequately root canal treatment were significantly higher than inadequately root canal-treatment, regardless of the quality or presence of the coronal restoration (P < .001).

**Table 2:** An overview of the relationship between the quality of root filling, coronal restorations and periapical status (n = 1014)

	healthy (pai 1)	apical periodontitis (pai 2-5)	total
**coronal restoration**			
adequate	183 (59%)	127 (41%)	310 (30.6%)
inadequate	143 (20.3%)	561(79.7%)	704 (69.4%)
total	326 (32.1%)	688 (67.9%)	1014
root filling			
adequate	254 (59.8%)	171(40.2%)	425 (41.9%)
inadequate	72 (12.2%)	517 (87.8%)	589 (58.1%)
total	326 (32.1%)	688 (67.9%)	1014

For the 310 (30.6%) teeth with adequate coronal restorations, the success rate was 59%. The group with inadequate restorations consisted of 704 teeth (69.4%), and the success rate in this group was 20.3%. The difference between the 2 groups was statistically significant(P < .001).

The healthy rate was 76.2% (231/303) for cases with adequate root canal treatment and adequate restorations. In cases of adequate root canal treatment and inadequate restoration, the success rate was 45% (55/122) (Table 3). The difference between the 2 groups was statistically significant, indicating that the outcome of adequately treated root canals was affected by the quality of the coronal restoration (P = .04).

**Table 3:** The periapical status for combinations of different qualities of coronal restoration and root canal fillings (n = 1014)

coronal restoration	root filling	n	healthy (pai 1)	%
adequate	adequate	303	231	76.2
inadequate	adequate	122	55	45.0
adequate	inadequate	231	25	10.8
inadequate	inadequate	358	15	3.7

Teeth with inadequate treatment and adequate restoration showed a success rate of 10.8% (25/231). Teeth with inadequate treatment and inadequate restoration yielded the lowest healthy rate of this study, i.e., 3.7% (15/358). Statistical analysis revealed that the healthy rate of inadequate root canal treatment was also significantly affected by the quality of coronal restorations. When compared to teeth with inadequate root canal treatment and adequate restoration, the difference was statistically significant (P = .02).

## Discussion

This study is a cross-sectional study. The main disadvantages of a cross-sectional study are that the data analyzed are restricted to the information available and thereby vulnerable to biases of interpretation [16]. For instance, radiographs were examined at a given point in time, and it was impossible to determine whether a periapical lesion was healing or not. On the other hand, misinterpretations and misdiagnoses in cross-sectional studies are known to be fairly equally distributed, so the results are still meaningful [17]. Additionally, a large sample size and random selection are the most important advantages of this method.

In intraoral radiography, the 3-dimensional object is compressed into a 2-dimensional image from which the observer has to mentally recreate the 3 dimensions [18]. This can be difficult even when more than a single radiograph is used. The use of more than 1 radiograph is of course inestimable when there is a need to display the roots of a tooth with as little superimposition onto each other as possible and when root fractures are suspected [18]. Best possible periapical radiographs are obtained when the paralleling technique is used [19]. This is, however, not always possible, e.g., in the upper molar regions where, owing to anatomic conditions, there is the greatest need for 3-dimensional information. Therefore, periapical intraoral radiographs may be quite sufficient in many areas and less so in others, depending on the diagnostic problems and the anatomic conditions [18].

Another limitation of this and other studies with a similar methodology is the fact that radiographic evaluation without clinical data does not always provide reliable information. For example, leaky occlusal margins and cracks in restorations could not be observed in radiographs. Likewise, apical periodontitis lesions limited to the cancellous bone might pass unnoticed in a radiographic examination [20]. Moreover, the microbiologic conditions of the root canal system, which influence the treatment outcome, cannot be inferred on the basis of the radiographic examination [21]. The accuracy of panoramic radiographs in the detection of apical periodontitis has been also questioned although Ahlqwist et al [22] supported their use in epidemiological studies. This was also supported by Muhammed and Manson-Hing [23] who showed no statistically significant difference between the panoramic and periapical surveys in the detection of periapical changes. However, they also suggested that a complete radiographic examination should include periapical, panoramic and bite-wing radiographs. In this study, periapical radiographs were only used when panoramic radiographs with poor image quality particularly in the upper and lower anterior region. In addition, only teeth scored as PAI 1 were accepted as healthy. Further studies with more sensitive techniques, such as cone beam computed tomography, have the potential to minimize this limitation.

This study of Turkish patients living in rural areas has similar results for the prevalence of periapical lesions to that of other epidemiological studies in Turkey and other countries [6,8-10,12,24-32]. In relation to these results, it is evident that a need for betterment of the quality of root canal treatment in general dental practice. In fact, these studies have been performed on people who live in cities and have high incomes and more treatment opportunities. Studies from controlled environments are usually carried out by specialists or supervised operators and reveal the potential outcome of root canal treatment rather than its realistic outcome in the general population. In Turkey, almost 17.1 million people are living in rural areas. Prevalence of perapical lesions in root-filled teeth in Turkey are conducted on people who live in cities and obtain medical treatment in private and public university hospitals [30,32] This research is the first in terms of targeting a population from a rural area. Kayahan et al. [32], an example of a study done in Turkey, detected 1268 teeth that received root canal treatment among 280 patients. In 754 (59.5%) of these teeth, there were no pathological findings. In another study by different researchers, the panoramic radiographs of 375 patients were investigated, and root canal treatment was applied to 449 teeth [30]. In 209 (46.5%) teeth, there were no pathological findings. In our study, 688 (67.9%) of 1014 teeth underwent root canal treatment, and apical periodontitis was detected. The income and treatment opportunities for patients, especially those living in rural areas of Turkey, are limited. Perhaps this explains the high apical periodontitis rate found in our study.

Consequently, it was found that the prognosis of inadequate root canal treatment was not affected by coronal restoration. When root canal treatment was inadequate, there was only a 7.4% difference between the inadequate and adequate crown restoration groups. This shows that the quality of the root canal treatment is the most important factor affecting the health of peri-radicular tissues.

The results from the present study confirm the findings of other studies that found the quality of the root canal treatment to be a key factor for prognosis with or without adequate coronal restoration. Siqueira et al.[10] reported that if the root canal filling was inadequate, it did not matter whether the coronal restoration was adequate, inadequate, or absent; the tooth would still have a poor prognosis when compared to an adequately filled tooth. Tronstad et al.[11] suggested that a correlation exists between the quality of the restoration and peri-radicular health but concluded that the quality of the restoration was significantly less important than the quality of the root canal filling. Additionally, Kayahan et al.[32] reported that, in an urban Turkish subpopulation, healthy periapex rates were significantly higher in teeth with good root canal filling regardless of the type of restoration.

In the literature, there are different studies that discuss the quality of root canal treatment and the effects of coronal restorations on peri-radicular tissues. Moreover, they have different results.

Kirkevang et al.[25] stated that the combination of inadequate root canal treatment and inadequate coronal restoration was associated with an increased incidence of apical periodontitis. This result was also supported by Hommez et al.[33] who demonstrated that an adequate coronal restoration and adequate root canal treatment were both important to the overall success of the root canal treatment. Ray and Trope [12] suggested that the quality of the restoration had a greater impact on periapical health than the quality of the root canal filling. Tavares et al.[16] reported that higher success rates for teeth with adequate or inadequate root canal treatment were always observed in teeth with adequate coronal restorations. Ricucci et al.[34] reported that exposure of root fillings to the oral microbiota was not significantly correlated with peri-radicular status. In our study, when a coronal restoration was inadequate, the success rate of adequately treated canals was reduced. Laboratory studies have suggested that the direct exposure of a root canal filling to microorganisms and their products may facilitate reinfection of the root canal system in a relatively short time [35-37].

## Conclusions

Our results revealed a high prevalence of periapical lesions in root canal treatment teeth. The results from the present study confirm the findings of other studies that found the quality of the root canal treatment to be a key factor for prognosis with or without adequate coronal restoration.

## Competing interests

The authors declare that they have no competing interests.

## Authors' contributions

KG and HA made substantial contributions to the conception and design of this study, participated in data collection, statistical analysis and interpretation of results, drafted and revised the final manuscript, and read and approved the final manuscript. KO and KD participated in the design and data analysis. All authors read and approved the final manuscript

## Pre-publication history

The pre-publication history for this paper can be accessed here:

http://www.biomedcentral.com/1472-6831/11/20/prepub
